# Hippocampal CaMKII inhibition induces reactivation-dependent amnesia for extinction memory and causes fear relapse

**DOI:** 10.1038/s41598-023-48454-1

**Published:** 2023-12-07

**Authors:** Andressa Radiske, Carla Miranda de Castro, Janine I. Rossato, Maria Carolina Gonzalez, Martín Cammarota

**Affiliations:** 1 Memory Research Laboratory - Brain Institute, Federal University of Rio Grande do Norte, Natal, RN Brazil; 2 Edmond and Lily Safra International Institute of Neuroscience, Macaiba, RN Brazil; 3 Department of Biophysics and Pharmacology, Biosciences Center, Federal University of Rio Grande do Norte, Natal, RN Brazil; 4 Department of Physiology, Biosciences Center, Federal University of Rio Grande do Norte, Natal, RN Brazil

**Keywords:** Extinction, Hippocampus

## Abstract

Hippocampal GluN2B subunit-containing NMDAR (GluN2B-NMDAR) activation during recall destabilizes fear extinction memory, which must undergo brain-derived neurotrophic factor (BDNF)-dependent reconsolidation to persist. Ca2+/calmodulin-dependent protein kinase II (CaMKII) is a Ser/Thr protein kinase essential for hippocampus-dependent memory processing that acts downstream GluN2B-NMDAR and controls BDNF expression, but its participation in fear extinction memory reconsolidation has not yet been studied. Using a combination of pharmacological and behavioral tools, we found that in adult male Wistar rats, intra dorsal-CA1 administration of the CaMKII inhibitors autocamtide-2-related inhibitory peptide (AIP) and KN-93, but not of their inactive analogs scrambled AIP and KN-92, after fear extinction memory recall impaired extinction and caused GluN2B-NMDAR-dependent recovery of fear. Our results indicate that hippocampal CaMKII is necessary for fear extinction reconsolidation, and suggest that modulation of its activity around the time of recall controls the inhibition that extinction exerts on learned fear.

## Introduction

Single brief non-reinforced re-exposure to the training context or related cues destabilizes consolidated memories, which to persist must undergo reconsolidation, a protein synthesis-dependent restabilization process that may also update or strengthen them^1^. Instead, repeated or prolonged non-reinforced recall may cause memory extinction, a process that also requires brain protein synthesis but, unlike reconsolidation, induces a new association that decreases the amplitude or probability of emission of the original learned response, preventing it from continuing to control behavior^2^.

Recent research on memory reconsolidation and extinction has focused on their neurochemical and behavioral properties, but much less effort has been devoted to analyzing their interaction. On this point, we previously found that recall-induced upregulation of hippocampal GluN2B subunit-containing NMDAR (GluN2B-NMDAR) destabilizes fear extinction memory^3^, which must undergo brain-derived neurotrophic factor (BDNF)-dependent reconsolidation in dorsal CA1 to remain behaviorally available^4–6^.

Ca2+/calmodulin-dependent protein kinase II (CaMKII) is a crucial neuronal calcium effector^7^ directly associated with memory consolidation, extinction, and reconsolidation^8,9^. Indeed, CaMKII is highly enriched at the postsynaptic side of hippocampal glutamatergic synapses^10^, where it controls rapid excitatory neurotransmission as well as NMDAR-dependent synthesis of memory-relevant proteins^11^, including BDNF^12,13^. However, the contribution of hippocampal CaMKII to fear extinction reconsolidation has not yet been studied.

## Results

To analyze the possible involvement of hippocampal CaMKII in extinction memory reconsolidation, we implanted microinjection cannulas into the CA1 region of the dorsal hippocampus of adult male Wistar rats and trained them in step-down inhibitory avoidance (SDIA; 0.4-mA/2-s footshock), a fear-motivated learning task in which stepping down from a platform is paired with a footshock, inducing a long-lasting hippocampus-dependent aversive memory^14^.

**Figure 1:**
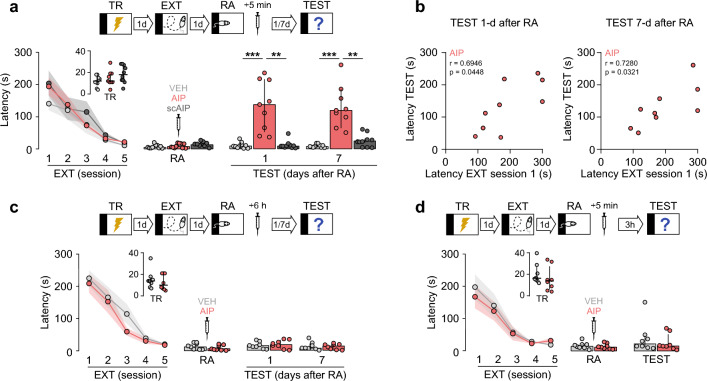
Early post-reactivation inhibition of dorsal-CA1 CaMKII impairs fear extinction memory and induces learned fear relapse. (**a**) SDIA-trained rats (TR) were subjected to 1 daily extinction session for 5 consecutive days (EXT; first session 1-d post-TR). One day after the last extinction session, fear extinction memory was reactivated (RA) and 5-min later, animals received bilateral intra-dorsal CA1 microinjections of VEH, AIP, or scAIP. Retention was evaluated 1-d and 7-d later (TEST). (**b**) Spearman correlation for AIP-treated animals showing step-down latencies during the first extinction session versus 1-d or 7-d TEST sessions. (**c**) Animals were treated as in A except that they received VEH or AIP 6-h post-RA. (**d**) Animals were treated as in A except that retention was evaluated 3-h post-RA.

**Figure 2:**
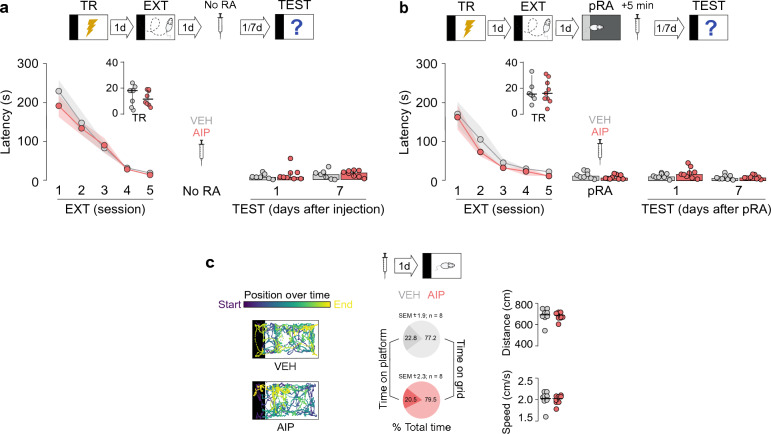
Inhibition of dorsal-CA1 CaMKII in the absence of extinction memory reactivation does not affect retention. (**a**) SDIA-trained rats (TR) were subjected to 1 daily extinction session for 5 consecutive days (EXT; first session 1-d post-TR). One day after the last extinction session, animals received bilateral intra-dorsal CA1 microinjections of VEH or AIP. Retention was evaluated 1-d and 7-days later (TEST). (**b**) Animals were treated as in A except that they received VEH or AIP 5-min after a pseudo-RA session (pRA) performed in a non-aversive box similar to the SDIA training box but painted gray and containing a platform made of transparent Plexiglass instead of wood. (**c**) Naive animals received bilateral intra-dorsal CA1 microinjections of VEH or AIP and 1-d later were allowed to freely explore the SDIA-training box for 300-s to evaluate locomotor activity. No footshock was given. Representative color traces show the animals’ position during the exploration session. Circular plots show the percentage of total exploration time animals spent on the platform and grid. Dot plots show distance travelled and speed during the exploration session.

**Figure 3:**
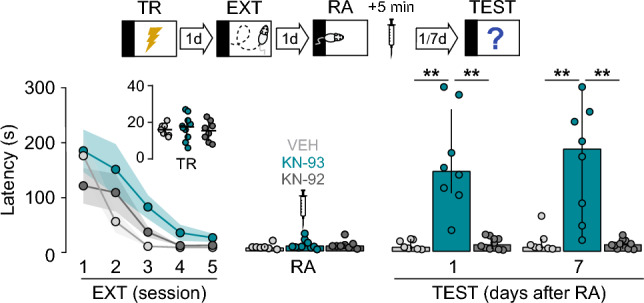
KN-93 mimics the effect of AIP on fear extinction memory. SDIA-trained rats (TR) were subjected to 1 daily extinction session for 5 consecutive days (EXT; first session 1-d post-TR). One day after the last extinction session, fear extinction memory was reactivated (RA) and 5-min later animals received bilateral intra-dorsal CA1 microinjections of VEH, KN-93, or KN-93 inactive analogue KN-92. Retention was evaluated 1-d and 7-d post-RA (TEST).

**Figure 4:**
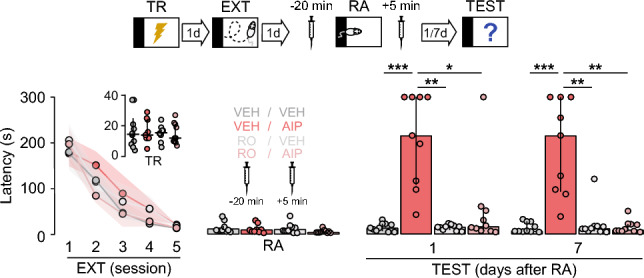
Pre-reactivation blockade of dorsal-CA1 GluN2B-NMDAR impedes the effect of CaMKII inhibition on fear extinction memory. SDIA-trained rats (TR) were subjected to 1 daily extinction session for 5 consecutive days (EXT; first session 1-d post-TR). One day after the last extinction session, animals received bilateral intra-CA1 microinjections of VEH or RO25‐6981, and 20-min later fear extinction memory was reactivated (RA). Five min thereafter, animals were given bilateral intra-dorsal CA1 microinjections of VEH or AIP. Retention was evaluated 1-d and 7-d post-RA (TEST).

Starting 1-d after SDIA training, and once a day for 5 consecutive days, we placed the animals back on the training-box platform and when they eventually stepped down from it, did not receive a footshock but were instead left to freely explore the training-box for 30-s, during which time they went up and down the platform several times. This procedure induces a clear-cut, long-lasting, hippocampus-dependent extinction memory that is not prone to renewal (i.e., the reappearance of the extinguished response induced by testing the subjects outside the extinction context^15^), spontaneous recovery (i.e., the reappearance of the extinguished response induced by the mere passage of time^16^), or reinstatement (i.e., the reappearance of the extinguished response induced by the non-contingent presentation of the unconditioned stimulus^17^)^5,18^. One day after the last extinction session, we subjected the animals to an extinction memory reactivation session (RA), and 5-min later they were given intra-dorsal CA1 microinjections of vehicle (VEH), the CaMKII substrate-competitive catalytic activity inhibitor AIP (10 nmol/side), or AIP inactive analog, scAIP (10 nmol/side). Retention was evaluated twice, 1-d and 7-d after RA. We found that AIP, but not scAIP, impaired extinction memory and recovered the fear response, which persisted for at least 7-d (Fig. 1a; TEST 1-d after RA: H = 17.29, *p* = 0.0002; *p* < 0.001 for VEH vs AIP, *p* < 0.01 for scAIP vs AIP; TEST 7-d after RA: H = 19.07, *p* < 0.0001; *p* < 0.001 for VEH vs AIP, *p* < 0.01 for scAIP versus AIP, in Dunn’s multiple comparisons after Kruskal–Wallis test). Non-parametric Spearman analysis for AIP-treated animals revealed a positive correlation in step-down latency between the first extinction session and the test sessions (Fig. 1b), suggesting that post-RA CaMKII inhibition indeed returned learned fear to pre-extinction levels. AIP did not affect retention when given 6-h post-RA (Fig. 1c), or when it was administered 5-min after RA, but extinction memory was tested 3-h instead of 1-d later (Fig. 1d). Furthermore, intra-CA1 AIP had no effect on extinction memory retention when given 1-d after the last extinction session but in the absence of RA (Fig. 2a), or 5-min after a pseudo-RA session performed in a non-aversive SDIA training box (Fig. 2b). AIP did not impair locomotor/exploratory activity when injected into the dorsal CA1 region of naive animals 1-d before a 5-min-long SDIA training-box free-exploration session (Fig. 2c). The effect of AIP on fear extinction was mimicked by the ca2+/calmodulin-competitive CaMKII catalytic activity inhibitor KN-93 (5 µg/side), but not by KN-93 inactive analogue KN-92 (5 µg/side; Fig. 3; TEST 1-d after RA: H = 15.84, *p* = 0.0004; *p* < 0.01 for VEH vs KN-93, *p* < 0.01 for KN-92 vs KN-93; TEST 7-d after RA: H = 13.50, *p* = 0.0012; *p* < 0.01 for VEH vs KN-93, *p* < 0.01 for KN-92 vs KN-93, in Dunn’s multiple comparisons after Kruskal–Wallis test). When given into dorsal CA1 20-min before RA, the GluN2B-NMDAR antagonist RO25‐6981 (2.5 μg/side), which impedes SDIA extinction memory destabilization without affecting recall or retention^3^, blocked the reappearance of the learned fear response triggered by AIP (Fig. 4; TEST 1-d after RA: H = 18.39, *p* = 0.0004; *p* < 0.001 for VEH/VEH vs VEH/AIP, *p* < 0.01 for RO/VEH vs VEH/AIP, *p* < 0.05 for RO/AIP vs VEH/AIP; TEST 7-d after RA: H = 19.34, *p* = 0.0002; *p* < 0.001 for VEH/VEH vs VEH/AIP, *p* < 0.01 for VEH/RO vs VEH/AIP, *p* < 0.01 for RO/AIP vs VEH/AIP, in Dunn's multiple comparisons after Kruskal–Wallis test).

## Discussion

Our results concur with reports suggesting that extinction does not delete memory but creates an inhibitory trace that competes with the original one for behavioral control. Our data also confirm that recall destabilizes fear extinction memory, which must be restabilized through reconsolidation to keep fear inhibited, and demonstrate that extinction memory reconsolidation requires hippocampal CaMKII. This claim is based on experiments showing that AIP and KN-93, but not their inactive analogues scAIP and KN-92, lastingly impaired extinction, inducing time-dependent recovery of learned fear, and is further supported by the fact that this relapse was not immediate but took time to occur and was prevented by impeding extinction memory destabilization via GluN2B-NMDAR blockade with RO25‐6981. In this regard, it is important to note that the CaMKII inhibitors we used in our experiments block CaMKII catalytic activity and therefore they also interfere with CaMKII binding to GluN2B-NMDAR, which could be more important for hippocampal synaptic plasticity than CaMKII enzymatic activity per se^19^. It has been suggested that impairing reconsolidation or enhancing memory extinction may help treat post-traumatic stress disorder (PTSD) patients^20,21^. However, despite some promising initial results, psychotherapeutic and pharmacotherapeutic interventions based on the modulation of extinction and reconsolidation mechanisms have been ineffective in quenching the persistent recollection of traumatic memories, either because these memories recover spontaneously after the completion of therapy or because inducing their recall is often unpractical or unethical, requiring the use of indirect or symbolic reminders unable to effectively trigger destabilization. In this regard, our findings not only corroborate the notion that reactivated memory processing dynamics are more complex than originally thought, suggesting the possibility that extinction memory is open to editing during recall, but also indicate that, through its interaction with GluN2B-NMDAR, CaMKII regulates the competition between antagonistic mnemonic representations and may be a suitable pharmacological target to treat PTSD. In agreement with this hypothesis, it has been recently reported that CaMKII is a potential molecular determinant of PTSD^22^, and that its modulation influences the interplay between extinction and reconsolidation and prevents the return of unwanted memories^23^.

## Methods

### Subjects

Experiments were performed in conformity with the USA National Institutes of Health’s Guide for Care and Use of Laboratory Animals and the ARRIVE guidelines. All procedures were approved by the local ethics committee (Comissão de Ética no Uso de Animais, CEUA, UFRN). Adult male Wistar rats (3-month-old; 300–350 g) were housed in groups of 5 per cage and maintained at 23 °C on a 12-h lights on/off schedule (lights on at 6:00 A.M.) with ad libitum access to food and water in the institutional vivarium. Experiments were performed during the light phase of the cycle and researchers were blinded to the animals’ treatment condition.

### Stereotaxic surgery

Rats were anesthetized with ketamine (80 mg/kg)/xylazine (10 mg/kg) and bilaterally implanted with 22-gauge stainless steel microinjection guides aimed to the CA1 region of the dorsal hippocampus (antero-posterior, − 4.2 mm; latero-lateral, ± 3.0 mm; dorso-ventral, − 3.0 mm from Bregma) using automated stereotaxic apparatuses. Meloxicam (0.2 mg/kg) was administered as an analgesic at the end of surgery. Animals were allowed to recover from surgery for at least 7-d before any other procedure.

### Drugs and microinjections

Myristoylated autocamtide-2-related inhibitory peptide (AIP)^24^ and myristoylated scrambled AIP (scAIP) were from FastBio (São Paulo, Brazil). KN-92 phosphate and KN-93 phosphate were from MedChemExpress (NJ, USA). RO25‐6981 was from Merck-Sigma Aldrich (São Paulo, Brazil). Drug and peptide doses were based on previous reports^3,25,26^. They were resuspended, aliquoted, and stored at − 20 °C upon arrival. Stock aliquots were diluted to working concentration in sterile saline (VEH) right before the experiments. Microinjections (1 µl/side) were carried out in a cleanroom adjacent to the experimental room through infusers fitted to the guide cannulas and connected to Hamilton syringes with tygon tubes. Flow rate (0.5 μl/min) was controlled using infusion pumps. After completing the microinjections, we left the infusers in place for 1-min to minimize backflow. Cannula placement was verified postmortem 1-d after the last behavioral test. Only data from animals with correct implants were analyzed.

### Step-down inhibitory avoidance (SDIA) training

Rats were handled once a day for 2 consecutive days prior to SDIA training, which was performed during the light phase of the subjective day in a 50 × 25 × 25-cm Plexiglas training box equipped with a grid floor through which scrambled footshocks could be delivered^27,28^. At the left end of the grid floor was a 5 × 8 × 25-cm wooden platform. For training, animals were placed on the platform facing the left rear corner of the training-box and, when they stepped down and placed all four paws on the grid, received a 0.4-mA 2-s scrambled footshock and were immediately removed from the training apparatus.

### SDIA memory extinction

To extinguish the SDIA response, beginning 1-d after SDIA training, rats were subjected to one daily extinction session for 5 consecutive days during which they were placed on the training-box platform and allowed to freely explore the apparatus for 30-s after stepping down to the grid. No footshock was delivered^29^. To reactivate SDIA extinction memory, animals were placed on the platform and after stepping down from it they were immediately removed from the training-box. No footshock was delivered^4^.

### Statistical analysis

The number of rats/group was based on previous reports. Rats were randomly assigned to experimental groups. Due to the 300-s ceiling imposed on latency and the fact that there is no validated multi-factor ANOVA for non-parametric data, latency was expressed as median ± interquartile range and analyzed by two-tailed Mann–Whitney U or Kruskal–Wallis test followed by Dunn’s post hoc comparisons using GraphPad Prism 10 (RRID:SCR_002798).

## Data Availability

The datasets supporting the findings reported in this study are available from the corresponding author upon reasonable request.
